# Chronic Gastritis*Read at meeting Travis County Medical Society, Austin, July, 1904.

**Published:** 1905-02

**Authors:** J. R. Hunter

**Affiliations:** Hornsby, Texas


### TEXAS MEDICAL JOURNAL.

ESTABLISHED JULY, 1885.

PUBLISHED MONTHLY.—SUBSCRIPTION $1.00 A YEAR.

VOL. XX. AUSTIN, FEBRUARY, 1905. No. 8.

### Original Contributions.

For Texas Medical Journal.

### Chronic Gastritis.*

BY J. R. HUNTER, M. D., HORNSBY, TEXAS.

At the last stated meeting of this society it was imposed upon me to prepare and read at tonight's meeting a paper on gastritis, and as all efforts on my part to shirk this task (for reasons already given) proved futile, I have decided to break the ice by giving you, as best I can, my ideas on chronic gastritis.

This, as you well know, is a chronic catarrhal inflammation or hyperemia of the mucous membrane of the stomach, associated with excessive mucous secretion and deranged gastric juice formation. The excessive mucus which is alkaline and very tenacious, is caused by the congestion of the blood vessels, thereby interfering with the maintenance of proper secretion and absorption. This mucus clings or adheres to the surface in such a way as to prevent the food from coming in direct contact with the mucous membrane of the stomach and thereby exciting the flow of gastric juice, as well as neutralizing it wholly or in part, and preventing it from reaching the food. Under such conditions, we could not reasonably expect anything other than the food to remain longer in the stomach than it should, where it undergoes maceration and fermentation, owing to the alkaline medium with which it comes in contact, which not infrequently develops large

*Read at meeting Travis County Medical Society, Austin, July, 1904.

quantities of gases (carbonic acid and marsh gas) that are belched up at intervals, with more or less discomfort.

The causes of chronic gastric catarrh are quite numerous, but some of the more important ones are alcohol, over-eating, too rapid eating, or imperfect mastication, irregular hours for meals, large amounts of ice water during meals, food which has undergone decomposition, and it is quite frequently secondary to other diseases, especially mitral disease of the heart, pulmonary affections, and interstitial hepatitis.

As symptoms, we have more or less loss of appetite, amounting sometimes to disgust for food, a bitter, unpleasant taste, a heavy-coated tongue, heartburn, which is due to the eructation of organic acids, nausea, sometimes vomiting, which is worse in the morning, headache, or a dull, unpleasant feeling in the head, vertigo, disturbed sleep, depression of spirits, generally constipation, and sometimes a feeling of distension or swelling of the stomach. We also find, in the form of reflex symptoms, more or less palpitation of the heart, irregular pulse, shortness of breath, the so-called stomach cough (which is more apt to be pulmonary than stomach), and as a rule no fever.

The disease is a very chronic one, and, if left to itself, finally becomes incurable, and may end in gastric ulcer, complete atrophy of the gastric tubules, and, it is claimed by some, pyloric thickening; however, I have never met the latter condition, or, at least, if I have, was unable to recognize it. With the evidence elicited by means of the test meal, for diagnostic purposes, I have had no personal experience, as the majority of my patients would almost as readily attempt to swallow a fence post as to consent to the use of the stomach pump. One of the greatest troubles I have ever met in the diagnosis was in a case of sarcoma of the stomach, accompanying the former condition, which was mistaken and treated for gastritis for some time. However, if one will bear in mind the general symptoms, the diagnosis may be pretty accurately arrived at.

Chronic gastric catarrh is a primary affection, and where the mucous membrane has not began to atrophy, can be cured by careful and persevering treatment, while, on the other hand, if it is secondary to mitral disease of the heart, pulmonary tuberculosis, or interstitial hepatitis, it can then be benefited only so far or in proportion to the improvement in the primary affection, and our prognosis in such cases should be very guarded, as the results are not always as brilliant as we would like them.

The first thing to be considered in the management of a case of gastritis, either acute or chronic, is to make very dilligent inquiry into the habits of the sufferer, which quite likely are acting as exciting causes, and without their correction all efforts upon our part will be rewarded by defeat. My experience with this class of work has led me to first endeavor to impress my patient that he must follow all instructions as nearly to the letter as possible, otherwise the beneficial effects of the treatment will be very limited. If the habit be formed of brooding over his condition, it is well to try to associate him with the most pleasant surroundings, also advise regular and systematic exercise, that should be fitted to each individual case.

For the thick and excessive mucus, which generally proves very troublesome, I am in the habit of ordering from one to two cups of warm water (as hot as can be borne) to be slowly sipped before meals, and especially before breakfast, as it undoubtedly aids in the dislodgement of the mucus and assists its escape into the duodenum, thereby partially cleansing the stomach, and exposing quite a number of the gastric tubules that would otherwise be occluded, and this is of no little advantage to the exit of the gastric juice. As to the frequency of feeding, I feel that no greater mistake can be made than to instruct our patients to take food in small quantities at short intervals, for by so doing we ignore the necessity of rest to the muscular coats of the stomach, and are thereby endeavoring to make it perform a task that even a healthy organ would fail under. My practice has been for some years to order my patients to take nourishment not oftener than six hours, unless it be in case of extreme emaciation, when it may be necessary to bring the interval to four hours, but never closer, and also discouraging the habit of taking large quantities of water. We should avoid, as far as possible, permitting ourselves to fall into the habit of giving a routine diet, for what will agree with one will disagree with another, and I feel it a much better practice to allow any and all foods that are not contra-indicated, or have proven uninjurious, with the exception of fats, sugar, pastries, and most fried dishes, which at the beginning I withhold, permitting them, as well as some of the alcoholic beverages, only in very rare cases, and then in very limited quantities.

Severe cases, and especially those complicating Bright's disease and cardiac troubles, will generally do better if we confine them for one or two weeks to an exclusive milk diet, the amount of which will depend, of course, upon our patient's size and strength, though two or three quarts per day will usually meet all indications, it being fresh and to be given warm and diluted with an equal volume of water, to which may be added salt ad libitum. Should much gastric irritation or nausea exist, it may be necessary to use skimmed milk or preferably buttermilk, the latter being taken undiluted.

As to other articles of food that may be utilized in these troubles, there are only a few to which I desire to call attention. These are oysters, which may be allowed either raw or broiled, butter sparingly, dried smoked beef, toast, a small piece of broiled steak, and rice, the steak being given more for its psychic effect on the flow of gastric juice than for its nourishing qualities, but should we have an excessive amount of hydrochloric acid, I would allow in connection with the above, and I believe it acts even better, lean roast beef, rare steak, bread and chicken, and after improvement is plainly noticeable, in either class, I permit the partaking of almost anything, preferably in small quantities and a general assortment, with instructions to exclude any that experience teaches the patient is harmful, laying particular stress upon pleasant surroundings while at meals, slow eating, and, above all, thorough mastication.

Turning to the medicinal side of the question, we find a very broad field confronting us, and one that will prove very perplexing to one seeking information on this line, but after a careful trial of quite a number I have found that by regulating the bowels with salines, calomel or preferably the F. E. Cascara Sagrada (P. D.), in from ten to twenty minim doses two or three times daily, one-half hour after meals, and giving hydrochloric acid, which I consider the most useful drug in these conditions, as it is pretty well settled that the efficiency of the gastric juice is due to this acid, and assuming that the hydrochloric acid is almost invariably diminished or absent (and I feel that most authors will sustain this view), and having in it not only a drug that possesses the power of converting the grandular pepsinogen in the protoplasm of the peptic cells into enzyme pepsin, but it also possesses the power of being a valuable antiseptic which will prevent the fermentation and decomposition that takes place in the stomach, and if permitted to continue will keep up the irritation. As to the quantity and mode of its administration (and I fear what the society will do for me in this connection will be plenty), I give from twenty to seventy minims of the dilute, in whole or divided doses, which should be much further diluted at the time of administration, and taken a short while after meals, and if in divided doses repeat every ten to fifteen minutes until the desired quantity is taken. If there is much uneasiness in the stomach, caused by the ingestion of food, I quite frequently order from one to four drams of the liquid Lactopeptine in a wineglass of water, to be taken as necessary. On the other hand, if my patient is unusually weak, I sometimes substitute the nitro-muriatic for the hydrochloric acid, giving it in connection with 1/50 to 1/20 grains of strychnine in wine of pepsin, and quite frequently use in connection with the above some one or combination of the bitter tonics before meals. Should, for any reason, the hydrochloric acid be objectionable, and some other form of treatment be necessary, I next resort to the nitrate of silver, which I give in capsule or pill form in from one-sixth to one-half grain doses one-half hour before meals, followed by a glass of warm water, and at times it may be advisable to resort to the bismuth preparations, although I consider them far less serviceable in the chronic form than in the ulcerative, at which times I give them in emulsion, and endeavor by position and exercise to bring them in contact with the entire mucus surface; though, should we have in the former condition an excess of acid or persistent vomiting, the bismuth in connection with cerium oxalate will prove useful, but unless they are positively indicated I withhold them from the fact that they are undoubtedly preparations that favor constipation, a condition we are endeavoring to relieve.

As to drugs to strengthen the weakened gastric and intestinal secretion, we may expect fair results from pancreatin, which I sometimes give in combination with soda, a short while after meals, generally employing it in tablet form, each of which represents five grains of the drug, giving from one to three tablets at a dose. With ptyalin and diastase, I have never been very favorably impressed, as I invariably get better results from pepsin, which may be given in from five to ten grain doses, and used as above stated.

The practice of lavage, which is so highly recommended by most all authors at the present date, has never impressed me favorably, for while there are some advantages and beneficial results to be derived from its use, there are also drawbacks to it that are not very easy to overcome, a few of which I shall mention, they being the ones most generally met with. First. The repugnance of the patient. Second. The unlikelihood of its proper employment by the patient. Third. The inaccuracy of the solution caused by the patient's negligence in their preparation. Fourth. The absolute impossibility of the physician being able to use the irrigation himself at all times and in all cases. Fifth. The likelihood of an additional irritation being set up by its use.

Fearing that some and probably all present may fail to see anything of value in this paper, for I fully realize that there is room for improvement, I shall call especial attention to a few ideas that I have endeavored to present. They are as follows: The probable error in diagnosis, the pleasant surroundings at meals, slow eating, thorough mastication, the absolute uselessness of endeavoring to convert our patient's stomach into an apothecary shop by giving a little of everything that we see recommended for such troubles, and last, that even in extreme atrophy of the tubules, if we can by any means restore motor power to the stomach and preserve the pancreatic activity, our patients will almost in every instance regain and maintain their health, though the secretion of the stomach be totally abolished.

